# DSP107 combines inhibition of CD47/SIRPα axis with activation of 4-1BB to trigger anticancer immunity

**DOI:** 10.1186/s13046-022-02256-x

**Published:** 2022-03-14

**Authors:** Ewa Cendrowicz, Lisa Jacob, Shirley Greenwald, Ami Tamir, Iris Pecker, Rinat Tabakman, Lucy Ghantous, Liat Tamir, Roy Kahn, Jasmine Avichzer, Alexandra Aronin, Shira Amsili, Elina Zorde-Khvalevsky, Yosi Gozlan, Martijn Vlaming, Gerwin Huls, Tom van Meerten, Michal Elhalel Dranitzki, Adam Foley-Comer, Yaron Pereg, Amnon Peled, Ayelet Chajut, Edwin Bremer

**Affiliations:** 1 University of Groningen, University Medical Center Groningen, Department of Hematology, Hanzeplein 1, 9713 GZ Groningen, The Netherlands; 2 Kahr Medical Ltd, 1 Kiryat Hadassah POB 9779, 9109701 Jerusalem, Israel; 3 Departments of Nephrology and Hypertension, Hadassah Medical Center, Faculty of Medicine, Hebrew University, Jerusalem, Israel; 4 Goldyne Savad Institute of Gene Therapy, Hebrew University Hospital, Jerusalem, Israel

**Keywords:** B cell lymphoma, CD47-SIRPα ‘don’t eat me’ signaling, Phagocytosis, T cell co-stimulation, 4-1BB

## Abstract

**Background:**

Treatment of Diffuse Large B Cell Lymphoma (DLBCL) patients with rituximab and the CHOP treatment regimen is associated with frequent intrinsic and acquired resistance. However, treatment with a CD47 monoclonal antibody in combination with rituximab yielded high objective response rates in patients with relapsed/refractory DLBCL in a phase I trial. Here, we report on a new bispecific and fully human fusion protein comprising the extracellular domains of SIRPα and 4-1BBL, termed DSP107, for the treatment of DLBCL. DSP107 blocks the CD47:SIRPα ‘don’t eat me’ signaling axis on phagocytes and promotes innate anticancer immunity. At the same time, CD47-specific binding of DSP107 enables activation of the costimulatory receptor 4-1BB on activated T cells, thereby, augmenting anticancer T cell immunity.

**Methods:**

Using macrophages, polymorphonuclear neutrophils (PMNs), and T cells of healthy donors and DLBCL patients, DSP107-mediated reactivation of immune cells against B cell lymphoma cell lines and primary patient-derived blasts was studied with phagocytosis assays, T cell activation and cytotoxicity assays. DSP107 anticancer activity was further evaluated in a DLBCL xenograft mouse model and safety was evaluated in cynomolgus monkey.

**Results:**

Treatment with DSP107 alone or in combination with rituximab significantly increased macrophage- and PMN-mediated phagocytosis and trogocytosis, respectively, of DLBCL cell lines and primary patient-derived blasts. Further, prolonged treatment of in vitro macrophage/cancer cell co-cultures with DSP107 and rituximab decreased cancer cell number by up to 85%. DSP107 treatment activated 4-1BB-mediated costimulatory signaling by HT1080.4-1BB reporter cells, which was strictly dependent on the SIRPα-mediated binding of DSP107 to CD47. In mixed cultures with CD47-expressing cancer cells, DSP107 augmented T cell cytotoxicity in vitro in an effector-to-target ratio-dependent manner. In mice with established SUDHL6 xenografts, the treatment with human PBMCs and DSP107 strongly reduced tumor size compared to treatment with PBMCs alone and increased the number of tumor-infiltrated T cells. Finally, DSP107 had an excellent safety profile in cynomolgus monkeys.

**Conclusions:**

DSP107 effectively (re)activated innate and adaptive anticancer immune responses and may be of therapeutic use alone and in combination with rituximab for the treatment of DLBCL patients.

**Supplementary Information:**

The online version contains supplementary material available at 10.1186/s13046-022-02256-x.

## Background

The mainstay of treatment for B cell non-Hodgkin lymphoma (NHL) is chemotherapy combined with CD20 monoclonal antibodies [1]. However, a subset of patients is refractory to treatment or will, after experiencing a complete response (CR), develop resistance to treatment and relapse with a poor prognosis [2]. Therefore, additional therapeutic options are needed. In this respect, combination therapy with the CD47 mAb Hu5F9-G4 (magrolimab) and CD20 antibody rituximab (RTX) yielded high CR rates in refractory/relapsed DLBCL patients in a phase Ib trial [3].

CD47 binding to its ligand SIRP-alpha (SIRPα) on phagocytes transmits a ‘don’t eat me’ signal that inhibits phagocytosis and, thereby, negatively regulates innate immunity [4, 5]. Many types of cancer overexpress CD47 to escape innate immunity [6], with the expression of CD47 often correlating with poor patient survival and poor response to standard therapy [7–9]. Various therapeutics that block CD47:SIRPα interaction, including CD47 mAbs (e.g., magrolimab) and fusion proteins comprising soluble SIRPα (e.g., TTI-621/2) [3, 10, 11], have promising clinical activity and are currently in Phase 2 or 3 clinical evaluation [3, 10–13].

Notably, treatment with CD47 antagonists also leads to antigen-presentation and cross-priming of T cells [14, 15]. Indeed, T cell cross-priming was even required for the therapeutic activity of a CD47 mAb in murine models, with no effect in T cell-deficient mice and CD8^+^ T cell depletion abrogating the effect in wild-type mice [16]. Thus, a clear rationale exists for developing a novel therapeutic that exploits CD47 checkpoint inhibition while simultaneously stimulating anticancer T cell immunity. An attractive target in this respect is the Tumor Necrosis Factor (TNF) receptor superfamily member 4-1BB (CD137), expression of which is selectively induced upon T cell receptor/major histocompatibility complex (TCR/MHC) interaction [17]. 4-1BB is considered a surrogate marker for the tumor-reactive T cell subset of Tumor-Infiltrating Lymphocytes (TILs) in the tumor microenvironment (TME) [18–20], whereas it is not expressed on resting T cells in peripheral blood [18]. Correspondingly, ex-vivo treatment with 4-1BB antibody enhanced expansion and reduced T cell exhaustion in TIL cultures for adoptive TIL therapy in many cancer types [21–24].

Treatment with 4-1BB antibodies also had strong antitumor activity in preclinical studies. However, in clinical trials, treatment was associated with toxicity and poor efficacy [20], which may partly be attributed to the signaling requirements of 4-1BB. Specifically, agonistic antibodies only effectively activate 4-1BB upon Fc-mediated cross-linking by Fc-Receptor (FcR)-positive effector cells [25]. Thus, efficacy is dependent on the presence of FcR-positive cells in the tumor. At the same time, FcR-mediated cross-linking is also held responsible for off-tumor toxicity, such as the liver toxicity encountered with 4-1BB antibody urelumab [17]. In a similar fashion, soluble trimeric 4-1BBL is ~ 100-fold less potent than oligomerized 4-1BBL in activating 4-1BB signaling [26]. Notably, this cross-linking requirement of 4-1BB has been therapeutically exploited using 4-1BBL fusion proteins that comprise a tumor-targeting domain that enables selective cross-linking of 4-1BB at the tumor [26, 27].

Here, we report on a new immunotherapeutic, termed Dual Signaling Protein 107 (DSP107), comprising the human extracellular domain (ECD) of SIRPα genetically fused to the human ECD of 4-1BBL. DSP107 assembles into a natural homotrimer due to the 4-1BBL trimerization domain and, upon binding to CD47 on cancer cells, breaks the CD47:SIRPα interaction. Simultaneously, CD47-mediated surface immobilization of DSP107 enables the delivery of the 4-1BBL:4-1BB costimulatory signal to tumor localized T cells. This dual immunomodulatory effect of DSP107 is designed to unleash both innate and adaptive immune responses at the tumor site.

## Materials and methods

A full reagent and cell line list with, methods of isolation and culturing of immune cells and cell lines and DSP107 production and characterization are listed in the supplementary materials and methods.

### Macrophage-mediated phagocytosis

M1 or M2 macrophages (1 × 10^4^ cells/well) were pre-seeded in 96-well plates for 24-48 h and CFSE-labelled cancer cells were subsequently added at an Effector: Target (E:T) ratio of 1:5 for 3 h at 37 °C. Where indicated, cancer cells were pre-incubated with DSP107, CD47 Ab (InhibRx), or SIRPαFc (TTI-622) at a concentration of 10 nM, with or without 1 μg/mL RTX. Subsequently, non-adherent DLBCL cells were removed by gently washing twice with RPMI 10% FCS. For microscopy analysis, macrophages were stained with CD11b-Alexa Fluor 594 antibody. Phagocytic uptake was assessed using the Life Cell Analysis System IncuCyte® (Sartorius) by quantifying the number of macrophages containing CFSE-labelled cancer cells per 100 macrophages. In addition, the number of cancer cells phagocytosed per macrophage, termed phagocytic index, was calculated by dividing the number of phagocytosed DLBCL cells by the total number of CFSE^+^ macrophages. Each condition was quantified by evaluating three randomly chosen fields of view. Confocal videos were generated using Zeiss Cell Discoverer 7 with LSM900 confocal head. For analysis of phagocytosis using flow cytometry, macrophages were detached using Trypsin/EDTA and stained with CD11b-APC antibody. Phagocytosis was quantified as the percentage of CFSE-positive CD11b^+^ macrophages. For long-term co-culture, CFSE-labelled SUDHL10 cells were added to 1 × 10^4^ pre-plated M1 macrophages at different densities (5, 2,5 or 1,25 × 10^3^ cells/well). To correct for the impact of unspecific cell-cell adhesion, control macrophages were fixed with methanol for 30 min at RT and subsequently mixed with cancer cells. After 72 h, cancer cells were removed from the plate, and the total number of remaining cells was counted using flow cytometry with counting beads.

### PMN-mediated trogocytosis

For PMN–mediated trogocytosis human granulocytes were mixed with CFSE-labelled tumor cells at a 1:1 ratio and treated with 10 nM of DSP107, CD47 Ab InhibRx, SIRPαFc, or His-SIRPα in the presence or absence of RTX (0,5–1 μg/mL) for 2 h at 37 °C. Trogocytosis was measured using flow cytometry and quantified as the percentage of CFSE-positive PMNs. To evaluate apoptosis induction in remaining cancer cells, samples were stained with AnnexinV-APC according to manufacturer instructions and the percentage of AnnexinV-positive cancer cells was evaluated using flow cytometry. Cooperativity index was calculated using formula: [(trogocytosis by single DSP107 treatment + trogocytosis by single RTX treatment)/ combination treatment].

### 4-1BB activation

To evaluate 4-1BB activation, the HT1080.4-1BB reporter cell line, which secretes Interleukin-8 (IL-8) upon activation of 4-1BB, was added to a 96-well plate pre-coated with human His-tagged recombinant CD47 (for 3 h at 5 μg/mL) and treated with a dose-range of DSP107 or mock-DSP for 24 h. Mixed culture experiments of HT1080.4-1BB and CHO-K1.wt or CHO-K1.CD47 clones were performed by co-plating cells at indicated ratios. Here, CHO.K1 cells were pre-treated with DSP107 for 1 h. IL-8 secretion in the supernatant was detected using the human IL-8 ELISA Kit according to the manufacturer’s recommendations. IL-8 levels were normalized, with 100% being defined as the highest IL-8 concentration detected per experiment.

For costimulation experiments with blood-derived T cells, isolated T cells were stained with CPDe450 and seeded in a 96-well plate coated with CD3 antibody (3 h at 0,5 μg/mL CD3 Ab, 1 μg/mL BSA) and treated with DSP107 (0.15 μg/mL) or CD28 antibody (2 μg/mL) for 96 h. For analysis, T cells were stained with viability dye, CD25-PE and 4-1BB-APC antibody whereupon the percentage of CD25+/41BB+ cells was determined within the viable CPDe450-labelled T cells using flow cytometry.

### T cell cytotoxicity

CD3^+^, CD4^+^ and CD8^+^ cells were isolated from PBMCs using negative selection magnetic beads. SC-1.scFvCD3 cells were stained with CytoLight Red, whereupon 2 × 10^4^ SC-1.scFvCD3 cells were mixed with T cells at indicated effector-to-target ratios. Co-cultures were incubated for 48–72 h with or without DSP107. Subsequently, cells were harvested and stained using AnnexinV-FITC and analyzed for cell death within the CytoLight Red positive SC-1.scFvCD3 population using flow cytometry. A similar setup was used for CHO.scFvCD3 and CHO.CD47.scFvCD3 cells with the following changes: cells were stained with CFSE and co-cultured with CD3^+^ cells in an ultra-low adhesion U-bottom plate. For analysis, cells were stained with AnnexinV-APC.

### B cell lymphoma xenograft in a humanized mouse model

The study was conducted at the Authority of Biological and Preclinical Models, the Hebrew University of Jerusalem, Sharet Specific Pathogen-Free (SPF) Unit under the Hebrew university ethic committee board approval (number MD-19-15,821-5). Fourteen-week female NSG mice were subcutaneously (s.c.) implanted with 1 × 10^7^ human SUDHL6 cells on day 0 of the study. On day 7, mice were randomly assigned to treatment groups. Mice were intravenously (i.v.) inoculated with 1 × 10^7^ PBMCs and treated with either intraperitoneal (i.p.) (150 μL/dose) PBS (*n* = 5 mice) or 250 μg/150 μL DSP107 (*n* = 6 mice) every other day starting from day 7 for 6 consecutive treatments. A second boost of previously frozen PBMCs from the same donor sample was administered on day 13. Tumor volume (mm^3^) was assessed on days 13, 15, and 20 using a microcaliper and was calculated with the following equation: (Width^2^ x Length/ 2). On day 22, mice were weighed, sacrificed, and tumor and spleen weights were measured. Formalin-Fixed-Paraffin-Embedded (FFPE) blocks from tumor tissues were generated (service was provided by GeneTherapy Institute, Hadassah Medical Hospital, Jerusalem). T cell staining was performed by Immunohistochemical (IHC) staining service of Gavish Research Services. In brief, IHC staining was performed on 4 μm FFPE sections using the Leica Bond max system (Leica Biosystems Newcastle Ltd., UK). Slides were baked for 120 min at 60 °C, dewaxed and pretreated with epitope-retrieval solution (ER, Leica Biosystems Newcastle Ltd., UK) followed by incubation for 30 min with primary antibodies for CD3+, CD8+, CD4+ and FOXP3+ T cells. The Leica Bond Polymer Refine Red kit (Leica Biosystems Newcastle Ltd., UK) was used for the FOXP3, CD8 and CD4 detection and the Leica Bond Polymer Refine HRP kit (Leica Biosystems Newcastle Ltd., UK) was used for CD3 detection. All slides were counter-stained with Hematoxylin. Analysis of IHC pictures was done using ImageJ.

### Nonhuman primate studies

The safety of DSP107 was tested under a Good Laboratory Practise (GLP) toxicology study. Naive cynomolgus macaques (16 males and 16 females) were given DSP107 by i.v. infusion twice a week, for 2 consecutive weeks (total of 4 infusions), at doses of 5, 15, and 50 mg/kg. All studies were conducted at Charles River Laboratories (Reno, Nevada) in accordance with Institutional Animal Care and Use Committee (IACUC) guidelines. As part of the comprehensive safety analysis, blood samples were collected for hematology and clinical chemistry before the treatment and at multiple time points after treatment. At the end of the study, gross necropsy, organ weight and histopathology of the full list of organs was conducted on all animals.

### Statistical analysis

The effect of DSP107 and/or antibody on phagocytosis or T cell-mediated cytotoxicity was determined by paired Student’s T-test using GraphPad Prism (GraphPad Prism; GraphPad Software, La Jolla, CA). The effect of DSP107 on tumor development in mice was evaluated using GraphPad Prism for unpaired t-tests. Statistical analysis on IHC was performed using the homoscedastic test. Where indicated * = *P* < 0.05; ** = *P* < 0.01; *** = *P* < 0.001.

## Results

### DSP107 forms a stable trimer and interacts with CD47 and 4-1BB

**Fig. 1:**
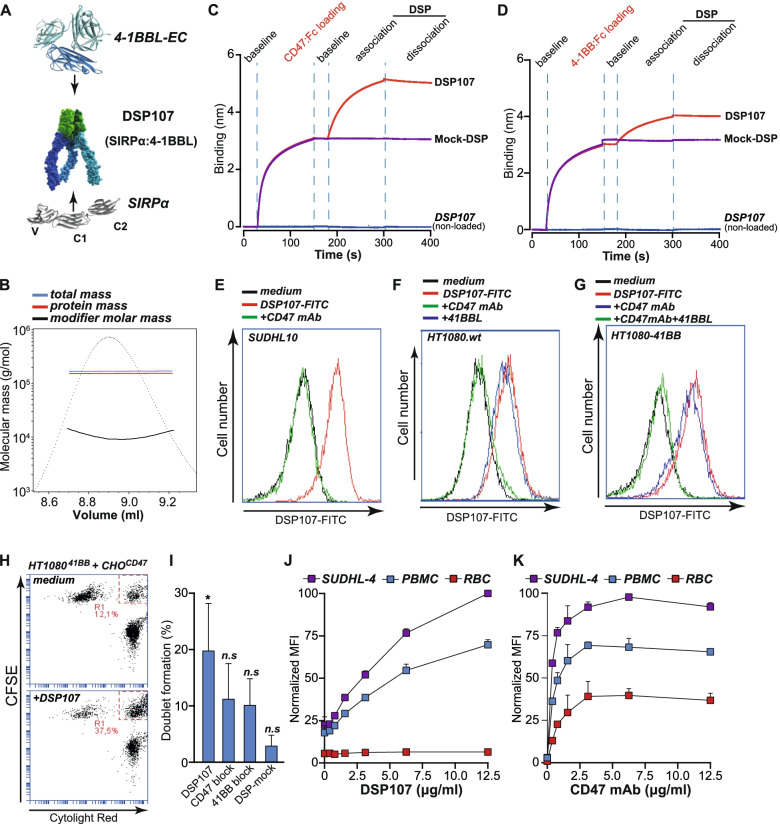
DSP107 *structure and binding characteristics* **A**) 3D schematic representation of trimeric DSP107 comprising genetically fused ECDs of human SIRPα (blue) to 4-1BBL (green). 3D structures are from Protein Data Bank and the 3D model was obtained based on PDB structures (4-1BBL PDB IDs: 2X29, 6FIB, 6BWV, 6CPR, 6D3N, 6CU0, 6MGE, 6MGP, 6A3V; SIRPα PDB IDs: 2JJS, 2JJT, 2UV3, 2WNG, 4CMM, 4KJY, 6BIT. **B**) SEC-MALS analysis of purified DSP107 showing the protein forms into a trimer in solution. Calculated molar mass is 210 g/mol/ 190 kDa **C**) Binding of DSP107 (red) or a DSP-MOCK (purple), to CD47:Fc immobilized on the plate and measured by BLI. no binding is observed to a chip without CD47:Fc (blue) **D**) Binding of DSP107 (red) or a DSP-MOCK (purple) to 4-1BB:Fc immobilized on the chip or without 4-1BB:Fc as in C. **E**) Binding of DSP107-FITC to SUDHL10 cells (red). DSP107 binding was blocked by CD47 mAb (green), unstained cells (black) **F**) Binding of DSP107-FITC to HT1080.wt cells (red), was completely blocked by CD47 mAb (green) and not affected by s4-1BBL (blue). **G)** Binding of DSP107-FITC to HT1080.4-1BB cells (red), was partially blocked by CD47 mAb (blue) and completely blocked by both CD47 mAb and soluble 4-1BBL (green). **H**) Doublet formation of CFSE stained CHO.CD47 and CytoLightRed stained HT1080.4-1BB cells without DSP107 (top) or with 5 μg/ml of DSP107 (bottom). Doublets are detected as a double-positive FITC-APC signal (bottom, top right corner). **I**) Doublet formation with DSP107, DSP-MOCK and after blocking with CD47 mAb or soluble 4-1BBL, parametric paired t-test was used with respect to control sample versus treatment with DSP107/mock-DSP or blocking agnets, * *p* < 0.05; **J**) DSP107 binding to a mixture of PBMCs (blue), SUDHL4 cells (purple) and RBCs (red) determined by flow cytometry, **K**) Binding of CD47 mAb to the mixture as in J. Error bars present in the figures represent SD

DSP107 comprises the ECD of human SIRPα genetically fused to the ECD of human 4-1BBL (Table S1) and, upon production, assembled as a homotrimeric protein with a calculated MW of ~ 175 kDa (Fig. 1A). This feature was experimentally confirmed using SEC-MALS analysis, yielding an apparent MW of ~ 190 kDa (Fig. 1B). Subsequent dual-binding ELISA using human CD47:Fc protein as capture and human biotinylated-4-1BB protein as the detector confirmed the integrity of both components of DSP107 with an average EC50 of 0.94 nM with three separately produced batches of DSP107 (Fig. S1A). Using Biolayer Interferometry, rapid time-dependent binding of DSP107 was detected to a CD47:Fc-coated biosensor, whereas a Mock-4-1BBL-based DSP lacking a SIRPα domain did not detectably bind (Fig. 1C). Similarly, DSP107 did not bind to the sensor without pre-coating of CD47:Fc (Fig. 1C). Analogously, DSP107 but not a mock SIRPα-based DSP, time-dependently bound to a 4-1BB:Fc-coated biosensor (Fig. 1D). Using surface plasmon resonance, the K_D_ of DSP107 for human CD47 and 4-1BB was subsequently determined to be 1.17 nM and 0.7 nM, respectively. The K_D_ of DSP107 for cynomolgus CD47 and 4-1BB was determined to be 1.75 nM and 0.34 nM, respectively, whereas DSP107 did not bind to mouse CD47 and 4-1BB (Table S2).

In a cell-based setting, FITC-labelled DSP107 bound to CD47-positive SUDHL10 cells (Fig. 1E; black vs. red line), with the binding being inhibited upon preincubation with an epitope-competing CD47 mAb (Fig. 1E, green line). For HT1080.wt cells, a similar binding pattern was detected, with DSP107 binding being abrogated by CD47 mAb alone (Fig. 1F). However, the binding of DSP107 to HT1080.4-1BB cells, expressing both CD47 and 4-1BB, was effectively abrogated only by preincubation with both CD47 mAb and s4-1BBL (Fig. 1G; green line vs. red line). For its intended mode of action, DSP107 should simultaneously bind to CD47 and 4-1BB expressed on distinct cells. In line with this, incubation of mixed cultures of CHO-K1.CD47 and HT1080.4-1BB cells with DSP107 triggered the formation of ~ 25% of doublets as evaluated by flow cytometry, whereas Mock-DSP did not (Fig. 1H,I). The formation of doublets by DSP107 was inhibited by preincubation with either CD47 mAb or s4-1BBL (Fig. 1I).

For CD47 antagonist therapy, the binding to and subsequent depletion of erythrocytes is a potential safety concern. However, in a mixed culture of RBCs, PBMCs and DLBCL line SUDHL4, no binding of DSP107 to RBCs was detected, whereas dose-dependent binding was observed for both SUDHL4 cells and PBMCs (Fig. 1J). In contrast, an antagonistic CD47 antibody did dose-dependently bind to RBCs, with binding already evident at the lowest antibody concentration (Fig. 1K). Of note, the binding of DSP107 to PBMCs is likely caused by the interaction of the SIPRα subdomain with CD47 expressed on immune cells because 4-1BB is not expressed on resting immune cell subtypes present in PBMCs (Fig. S1D). Indeed, the binding of DSP107 to PBMCs was abrogated by pre-incubation with CD47 mAb alone (Fig. S1F).

### DSP107 augments macrophage-mediated phagocytosis of cancer cells alone and in combination with rituximab (RTX)

**Fig. 2:**
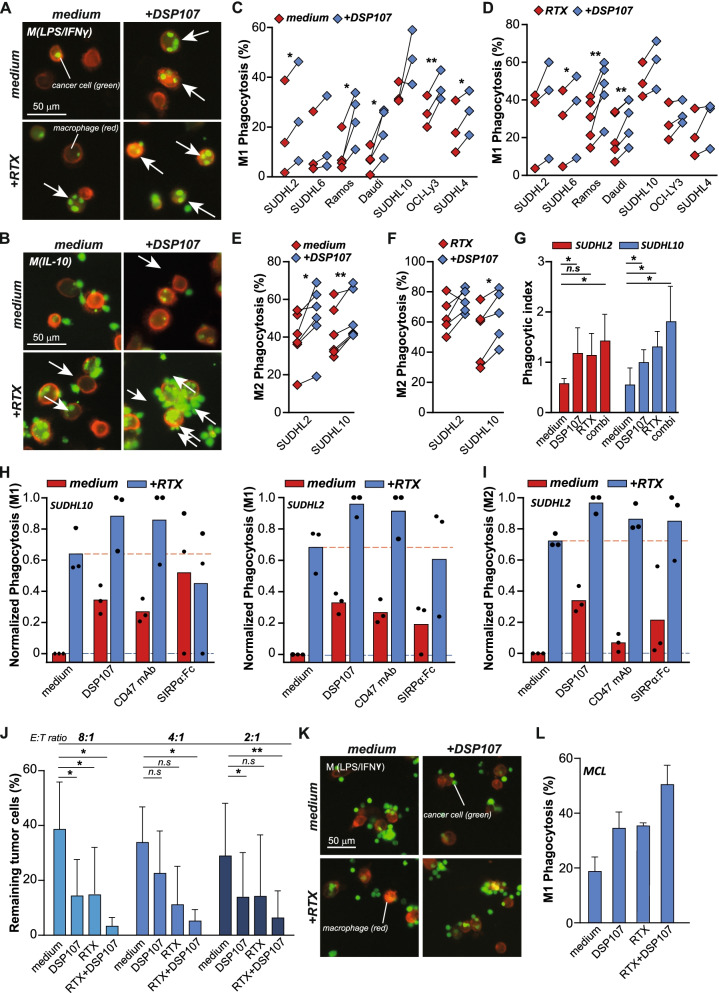
*DSP107 enhances macrophage-mediated phagocytosis of B cell lymphoma cell lines as a single agent and in combination with RTX.* **A**) Representative pictures of M1 macrophage-mediated phagocytosis of SUDHL10 cells untreated, treated with DSP107, RTX or RTX and DSP107. White arrows point macrophages with engulfed cancer cells, cancer cells (green) were labelled with CFSE and macrophages (red) were labelled with CD11b-AF594, scale bar 50 μm. **B**) Representative pictures of phagocytosis of SUDHL10 cells by M2c macrophages as in A. **C**) Phagocytosis of seven different B cell lines by allogeneic human M1 macrophages alone (red diamonds) or upon treatment with 10 nM DSP107 (blue diamonds). Each line represents the average phagocytic uptake calculated from 3 pictures of a single experiment, parametric paired t-test was used with respect to control sample versus each dose of DSP107, **D**) M1 macrophage-mediated phagocytosis of B cell lymphoma cells treated with 10 nM DSP107 and 1 μg/ml RTX (red diamonds) compared to RTX (blue diamonds) as in C, **E**) M2c-macrophage phagocytosis of SUDHL2 and SUDHL10 cells. Phagocytic uptake in the untreated sample (red diamonds) or upon treatment with 10 nM DSP107 (blue diamonds), parametric paired t-test was used with respect to control sample versus DSP107 treatment, **F**) Phagocytosis by M2c macrophages with 1 μg/ml RTX alone (red diamonds) and in combination treatment of 10 nM DSP107 and RTX (blue diamonds), as in E, **G**) Phagocytic index calculated for phagocytosis by M2 macrophages, parametric paired t-test was used with respect to medium control versus treatment with 10 nM DSP107, RTX or combination treatment, **H**) Comparison of DSP107, CD47 mAb and SIRPα:Fc, Phagocytosis assay by LPS/IFNγ stimulated macrophages on SUDHL10 (left) or SUDHL2 cells (right). DSP107, CD47 mAb or SIRPα:Fc for monotherapy (red bars) or combination treatment with RTX (blue bars) are used at an equal concentration of 10 nM. Phagocytosis results were normalized to the highest phagocytic uptake percentage of the same donor for a proper comparison of phagocytosis between the drugs. **I**) Comparison of DSP107, CD47 mAb and SIRPα:Fc for induction of phagocytosis by M2c macrophages. Results were normalized as in H. **J**) SUDHL10 cell removal by M1 macrophages after 72 h of co-culture, parametric paired t-test was used with respect to medium control versus treatment with DSP107, RTX or combination treatment. **K**) Representative pictures of phagocytosis with DSP107, RTX and combination of DSP107 and RTX of primary patient-derived MCL cells with autologous macrophages, cancer cells (green) were labelled with CFSE and macrophages (red) were labelled with CD11b-AF594, scale bar 50 μm. **L**) Analysis of primary malignant MCL cells by autologous macrophages (from one donor). Three pictures per condition were counted and phagocytosis was calculated as the number of cancer cells per 100 macrophages. Across the figure, t-test values: * = *p* < 0.05, ** = *p* < 0.01, *** = *p* < 0.001, all error bars in the figure represent SD

To delineate CD47 checkpoint activity of DSP107, fluorescently-labeled SUDHL10 cells were added to adherent M1 or M2c macrophages. Upon treatment of such a mixed culture with DSP107, SUDHL10 cells were clearly visible inside macrophages within 3 h, whereas no phagocytosed cells were detected in medium control (Fig. 2A,B). Similarly, a sub-optimal dose of RTX induced macrophage-mediated phagocytosis of SUDHL10 cells, but combined treatment with DSP107 and RTX led to the presence of multiple cancer cells inside most macrophages (Fig. 2A,B). In this time frame, the combination treatment also markedly enhanced the adhesion of cancer cells to macrophages (Fig. 2B, lower right panel). Upon quantification, phagocytosis of 5 out of 7 NHL cell lines by M1 macrophages significantly increased upon treatment with DSP107 compared to medium control (Fig. 2C), with RTX-mediated phagocytosis being augmented by DSP107 co-treatment (Fig. 2D). Similar pro-phagocytic activity of DSP107 alone or in combination with RTX was detected using M2c-like macrophages, as illustrated for SUDHL2 and SUDHL10 cells (Fig. 2E, F). Macrophage polarization to M1 or M2 subtypes was confirmed by flow cytometry by staining for CD86 (as M1 marker) and CD163 (as M2 marker) (Fig. S2). Subsequent assessment with confocal microscopy confirmed that cancer cells were phagocytosed and internalized by macrophages rather than being attached to macrophages (Video S1 for control macrophage and Video S2 for DSP107 treatment). Correspondingly, the phagocytic index was significantly increased upon treatment with DSP107 alone and in combination with RTX (Fig. 2G). Further, at equimolar concentrations, DSP107 treatment alone or in combination with RTX was equally effective in potentiating phagocytosis by M1-like and M2c-like macrophages as CD47 mAb and SIRPα:Fc (Fig. 2H, I). Moreover, the number of residual cancer cells upon DSP107 or RTX treatment alone was significantly reduced by ~50% compared to medium control after 72 h, with a further reduction down to ~10% residual cells upon combination treatment (Fig. 2J). Thus, DSP107 triggered prominent phagocytic removal of cancer cells by macrophages. Finally, in mixed cultures of primary patient-derived M1 macrophages and autologous MCL blasts, DSP107 treatment alone and in combination with RTX also significantly enhanced phagocytosis and triggered adhesion of cancer cells (Fig. 2K, L). Of note, although previously reported, no expression of 4-1BB was detected on any of the M1 or M2c macrophages (Fig. S2B). Therefore, DSP107 enhanced macrophage-mediated phagocytosis of B cell lines and primary B cell lymphoma blasts by blocking of CD47-SIRPα interaction. Further, DSP107 had comparable CD47 antagonist activity to a CD47 antibody and SIRPα:Fc.

### DSP107 triggers PMN-mediated trogocytosis of malignant B cells in-vitro

**Fig. 3:**
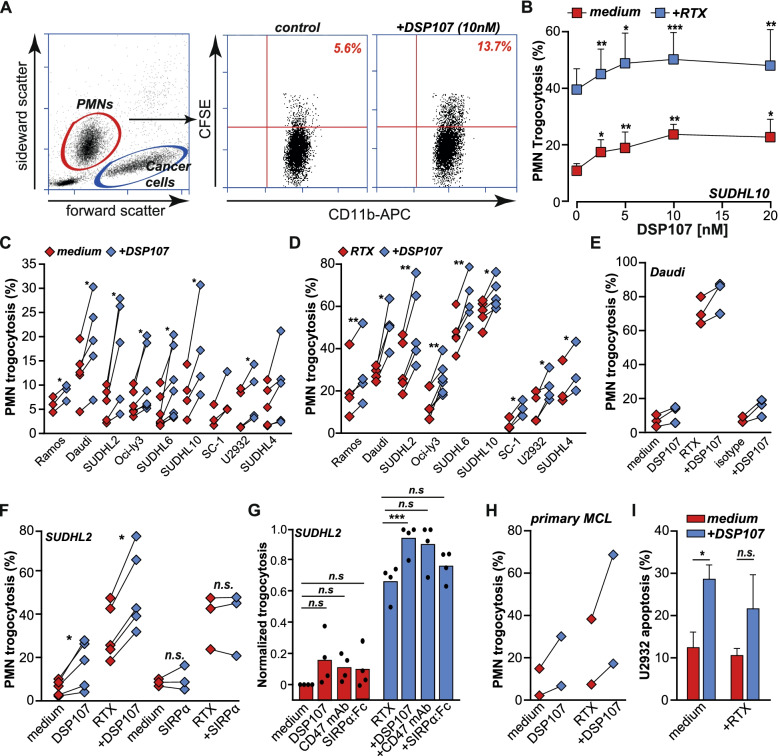
*DSP107 mediated PMN trogocytosis of B cell lymphoma cell lines*. **A**) Gating strategy for the PMN-mediated trogocytosis assay. PMNs are gated on forward-sideward scatter (left). Percentage of CD11b-APC labeled granulocytes positive for CFSE dye from cancer cells, calculated from the scatter (right), represents the level of phagocytic uptake. **B**) PMN-mediated trogocytosis with increasing concentrations of DSP107 (red) and combined with 1 μg/ml RTX (blue), parametric paired t-test was used with respect to control sample versus each dose of DSP107, **C**) PMN trogocytosis of nine B cell-lines without treatment (red) or with 10 nM DSP107 (blue). Each line represents a single experiment with an individual donor, parametric paired t-test, as described in B, was used, **D**) Comparison of PMN-mediated trogocytosis of nine B cell-lines between RTX (red) and combination treatment of RTX and DSP107 (blue). Analyzed as in B, **E**) PMN-mediated trogocytosis of Daudi cells with DSP107 and RTX or isotype control for RTX **F**) Comparison of 10 nM DSP107, comprising trimeric SIRPα (left) and 10 nM monomeric SIRPα (right) as prophagocytic agents. Analyzed as in B, **G**) Comparison of DSP107, CD47 mAb and SIRPα:Fc,. trogocytosis activity on SUDHL2 cells. DSP107, CD47 mAb or SIRPα:Fc for monotherapy (red) or combination treatment with RTX (blue) are used at an equal concentration of 10 nM. Trogocytosis results were normalized to the highest trogocytosis percentage of the same donor to compare the effect between drugs, parametric paired t-test was used with respect to control sample versus each drug. **H**) Trogocytosis of primary patient-derived malignant B cells by autologous PMNs from two donors **I**) AnnexinV staining of remaining cancer cells after trogocytosis assay, upon DSP107 stimuli or DSP107 with RTX (DSP107 bars) comparing to untreated cells or treatment with RTX alone (red bars), parametric paired t-test was used with respect to control sample versus treatment with DSP107. Across the figure, t-test values: * = *p* < 0.05, ** = *p* < 0.01, *** = *p* < 0.001, all error bars in the figure represent SD

CD47 also has previously been reported to negatively regulate PMN-mediated phagocytosis and trogocytosis. In line with this, a significant dose-dependent increase in PMN-mediated trogocytosis was detected upon DSP107 treatment in mixed cultures of CFSE-labeled cancer cells and PMNs (gating strategy in Fig. 3A), as illustrated for SUDHL10 (Fig. 3B). Treatment with 10 nM DSP107 induced a statistically significant increase in trogocytosis in seven out of nine NHL cell lines (Fig. 3C). Compared to treatment with RTX alone, the combination with DSP107 also significantly and dose-dependently increased trogocytosis in SUDHL10 (Fig. 3B). Correspondingly, a statistically significant effect of combination treatment was detected in all nine cell lines at 10 nM DSP107 (Fig. 3D), whereas control treatment with isotype antibody did not augment trogocytosis as illustrated for Daudi cells (Fig. 3E). The combination of RTX with DSP107 yielded a synergistic increase in trogocytosis as determined by cooperativity index (Fig. S3A), whereas treatment with a commercially available His-tagged ECD of SIRPα did not augment trogocytosis alone nor with RTX as illustrated for SUDHL2 cells (Fig. 3F). Further, compared to equimolar concentrations of CD47 mAb and SIRPα:Fc, DSP107 similarly induced trogocytosis alone and in combination with RTX (Fig. 3G). DSP107-mediated activation of PMN anticancer activity is due to CD47 checkpoint inhibitor activity, with no 4-1BB expression detected on PMNs (Fig. S3B). Interestingly, within the residual cancer cells after the trogocytosis experiment, a significant increase in apoptosis was detected upon DSP107 treatment from 16 to 28% (Fig. 3I). Similar treatment of cancer cells only with DSP107 did not trigger any increase in apoptosis (Fig.S3C), suggesting that DSP107 may activate PMN-mediate cytotoxicity. Finally, DSP107 also augmented trogocytosis of primary patient-derived MCL blasts by autologous PMNs by 6–15%, with a 10–25% increase when combined with RTX (Fig. 3H). Thus, DSP107 prominently activated PMN-mediated anticancer activity.

### DSP107 potentiates anticancer T cell activity

**Fig. 4:**
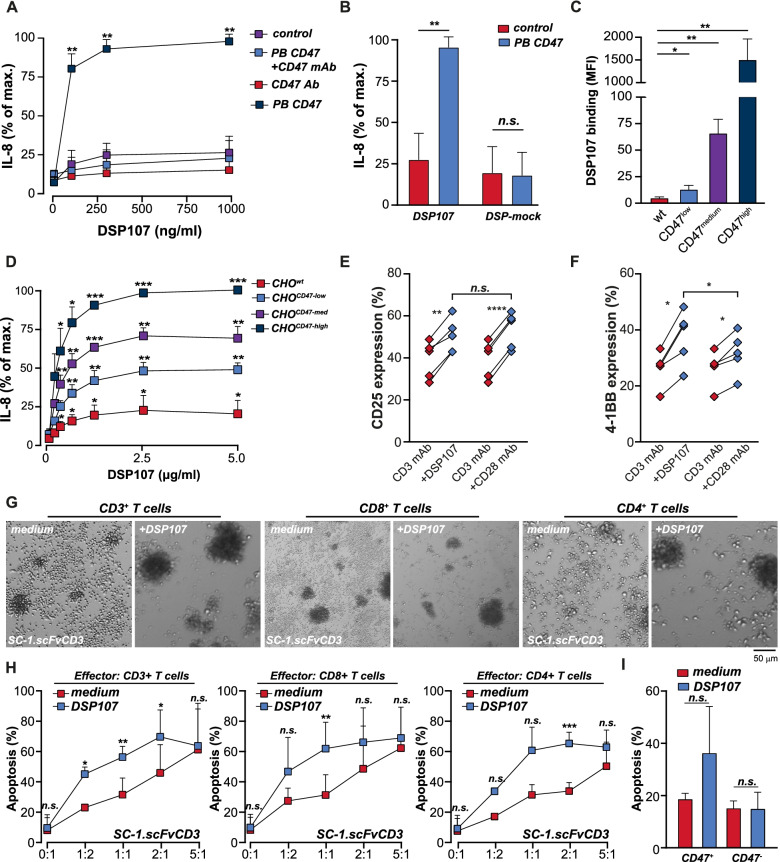
DSP107 induces T cell-mediated killing of tumor cells upon cross-linking via CD47 arm of DSP107. **A**) IL-8 release by HT1080.4-1BB cells triggered by increasing concentrations of DSP107 with CD47-coated plate (dark blue), when plate-bound CD47 is blocked with CD47 mAb (blue), without plate-bound CD47 or with CD47 mAb alone (purple and red), parametric paired t-test was used with respect to control sample (concentration 0) versus treatment with DSP107, **B**) IL-8 secretion upon treatment of HT1080.4-1BB cells with DSP107 or DSP-MOCK, containing 4-1BBL domain but lacking SIRPα domain, parametric paired t-test was used with respect to control sample treated with DSP107 or DSP-mock or treated sample with plated-bound CD47, **C**) Binding of DSP107 to CHO-K1 clones expressing low, medium and high levels of CD47 as measured by flow cytometry, parametric paired t-test was used with respect to control sample vs binding of DSP107, **D**) IL-8 secretion by HT080.4-1BB cells co-cultured with CHO-K1 clones expressing different levels of CD47 upon treatment with DSP107, analyzed as in C, **E**) Increase of CD25 expression on activated T cells (red diamonds) upon co-stimulation with DSP107 (blue diamonds, left graphs) or CD28 mAb (blue diamonds, right graph), paired t-test was used with respect to control sample (unstimulated) versus treatment with DSP107 or CD28 mAb **F**) Increase of 4-1BB expression on activated T cells (red diamonds) upon co-stimulation with DSP107 (blue diamonds, left graphs) or CD28 antibody (blue diamonds, right graph), paired t-test was used as in E), **G**) Representative pictures of co-cultures of SC-1.scFvCD3 with CD3^+^ (left), CD8^+^ (middle) and CD4^+^ (right) T cells, untreated (left picture in each panel) or upon treatment with DSP107 (right picture in each panel), scale bar: 50 μm. **H**) AnnexinV staining of residual SC-1.scFvCD3 cells in co-cultures with increasing amount of CD3^+^ (left), CD8^+^ (middle) or CD4^+^ (right) without treatment (red) or with 8,4 μg/ml DSP107 (blue), parametric paired t-test was used with respect to control sample (medium control – red) versus treatment with DSP107 (blue) at each E:T ratio. **I**) T cell-mediated killing of CHO^wt^scFvCD3 or CHO^CD47^scFvCD3 with and without DSP107 as measured by flow cytometry, parametric paired t-test was used with respect to control sample vs binding of DSP107. Across the figure, t-test values: n.s. = *p* > 0.05, * = p < 0.05, ** = p < 0.01, *** = p < 0.001, all error bars in the figure represent SD

DSP107-mediated activation of T cell costimulatory signaling by 4-1BB should only occur upon cross-linking after CD47-specific binding of DSP107. Indeed, incubation of HT1080.4-1BB cells with DSP107 on a CD47 pre-coated plate, but not on a non-coated plate, triggered an ~ 3–4 fold increase in IL-8 secretion (Fig. 4A). This IL-8 induction by DSP107 was abrogated by preincubation with epitope-competing CD47-blocking antibody (Fig. 4A) and was not detected upon incubation with a mock 4-1BBL-DSP lacking CD47-binding activity (Fig. 4B) nor by treatment of HT1080.wt cells (Fig. S4A). Further, in mixed cultures of HT1080.4-1BB cells with CHO-K1.CD47 clones that expressed different levels of CD47 (Fig. S4B), the level of binding of DSP107 to CHO-K1.CD47 (Fig. 4C) dictated the extent of IL-8 secretion by HT1080.4-1BB (Fig. 4D). Thus, 4-1BB activation by DSP107 clearly depended on initial CD47-specific binding and surface immobilization of DSP107. Of note, 4-1BB antibody treatment triggered IL-8 secretion by HT1080.4-1BB cells to a similar level as DSP107 upon cross-linking via plate-bound CD47 (Fig. S4C). Thus, 4-1BB antibody and DSP107 had similar potency in terms of 4-1BB costimulatory signaling. DSP107 also was able to co-stimulate T cells, with the percentage of CD25 and 4-1BB expressing T cells being significantly increased upon treatment with DSP107 (Fig. 4E and F, respectively). Notably, the increase in CD25 expression by DSP107 was comparable to treatment with CD28 agonistic mAb (Fig. 4E), but DSP107 induced significantly higher upregulation of 4-1BB compared to CD28 antibody treatment (Fig. 4F).

Since DSP107 potently triggered 4-1BB costimulatory signaling, its effect on T cell-mediated killing of cancer cells was assessed by co-culture of isolated CD3^+^, CD4^+,^ or CD8^+^ T cells with the CD47-positive NHL line SC-1.scFvCD3. This cell line was engineered to ectopically express a CD3 antibody fragment that triggers TCR signaling in an MHC-unrestricted manner. In such co-cultures, the formation of characteristic clusters of activated T cells was already detected in medium control (Fig. 4G, left panels). However, cluster formation was strongly potentiated by DSP107 treatment, with almost no individual T cell being detected (Fig. 4G, right panels). Correspondingly, a clear E:T ratio-dependent increase in apoptosis of SC1.scFvCD3 cells was detected in DSP107-treated mixed cultures with CD3^+^, CD4^+^, and CD8^+^ T cells (Fig. 4H). Of note, DSP107 had no single-agent activity towards SC1.scFvCD3 at E:T 0:1 (Fig. 4H). In similar mixed culture experiments with CHO.scFvCD3 cells, DSP107 treatment increased apoptosis compared to control culture only when CHO cells were additionally engineered to express human CD47 (Fig. 4I). Thus, DSP107 triggered 4-1BB-mediated costimulatory signaling and T cell-mediated anticancer activity only upon CD47-selective binding.

### DSP107 decreased tumor volume of SUDHL6 DLBCL tumors in NSG mice

**Fig. 5:**
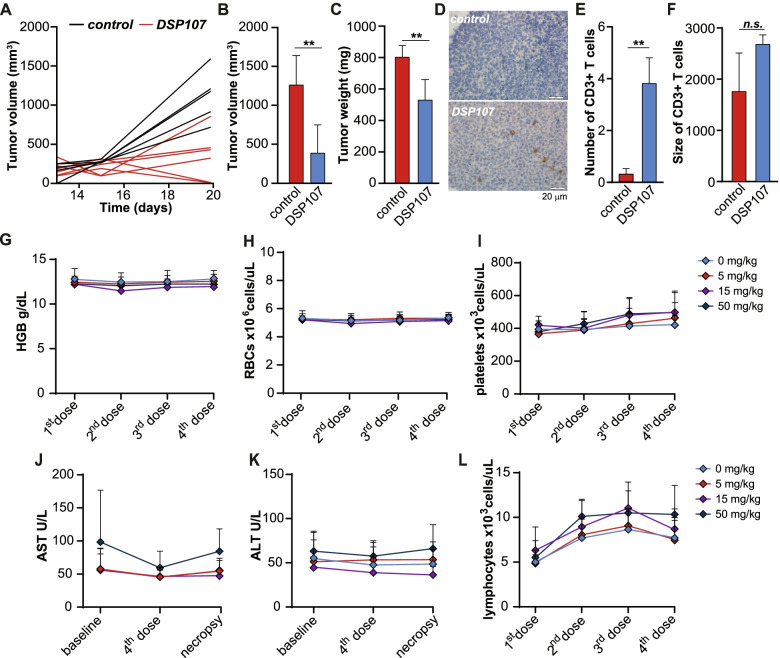
DSP107 reduces tumor growth in vivo and has good safety profile in non human primates. **A**) Size of the tumor for each individual mice. Five mice were in the control group and six mice were in the treatment group. **B**) Average tumor volume in the control and treatment group, **C**) Average tumor weight in the control and treatment groups. **D**) Immunohistochemistry staining of CD3^+^ T cells infiltrating the tumor in the control group (top) and the treatment of DSP107 (bottom), scale bar: 20 μm. **E**) the Average number of T cells in tumor tissue calculated from pictures in the control and treatment groups. **F**) Average size of T cells in the control and treatment groups. For all the mice Figs. (A-E), unpaired one sided t-test was used to compare T cell infiltration in the control and treatment groups n.s. = p > 0.05, error bars represent SD. **G**) Concentration of hemoglobin in the blood of cynomolgus monkey after injection of various doses of DSP107 (0, 5, 15 and 50 mg/kg), **H**) RBC count in blood of cynomolgus monkey after injection of various doses of DSP107 (0, 5, 15 and 50 mg/kg), **I**) Platelets count in blood of cynomolgus monkey after injection of various doses of DSP107 (0, 5, 15 and 50 mg/kg), **J**) AST level in serum of cynomolgus monkey after injection of various doses of DSP107 (0, 5, 15 and 50 mg/kg), **K**) ALT level in serum of cynomolgus monkey after injection of various doses of DSP107 (0, 5, 15 and 50 mg/kg), **L**) Total lymphocyte count in blood of cynomolgus monkey after injection of various doses of DSP107 (0, 5, 15 and 50 mg/kg). Across the figure, t-test values: n.s. = p > 0.05, * = p < 0.05, ** = p < 0.01, *** = p < 0.001, all error bars in the figure represent SD

To evaluate in vivo activity of DSP107, humanized NSG mice xenografted with SUDHL6 were reconstituted with human PBMCs (Fig. S5A). Treatment with DSP107 markedly inhibited tumor growth, with 2 out of 6 mice being tumor-free at the end of the experiment (Fig. 5A), an average 69% reduction in tumor volume (Fig. 5B) and 34% reduction in tumor weight (Fig. 5C) compared to control PBMC-injected mice. DSP107 treatment was further associated with a significant increase in CD3 + T cell infiltration (Fig. 5D, E) and a trend towards the larger size of infiltrating T cells (Fig. 5F). Additional IHC staining revealed that the main T cell type in the DSP107 treated group is CD4+ T cells (Fig. S5D, F), with very few and similar number of FOXP3+ T cells between the treated and the control groups (Fig.S5E, F). Similarly, low number of CD8+ T cells were found at identical levels between control and DSP107 treated groups (data not shown). Finally, DSP107 treatment did not negatively impact mouse or spleen weight (Fig. S5B, C).

### Safety of DSP107 in non-human primates toxicological study

Administration of DSP107 every 3 days for 4 doses (Days 1, 4, 7, and 10) by i.v. infusion was well tolerated in cynomolgus monkeys at levels of 5, 15 or 50 mg/kg/dose, with all monkeys surviving until scheduled study completion. Importantly, no DSP107-related adverse effects were observed on the hemoglobin (Fig. 5G), RBCs count (Fig. 5H), platelet count (Fig. 5I), coagulation, or blood chemistry parameters (Fig. 5J for liver enzyme AST and Fig. 5K for ALT). Moreover, no DSP107-related changes in the absolute count or relative percentages of T-lymphocyte subsets (T-helper, T-cytotoxic, or T-regulatory lymphocytes), B-lymphocytes, natural killer cells, or monocytes (see Fig. 5L for total lymphocyte counts). Further, no macroscopic, organ weight or microscopic changes related to DSP107 were observed in the organs of any of the monkeys.

## Discussion

Here, we report on the preclinical validation of DSP107 (SIRPα-41BBL) as a novel immunotherapeutic that combines innate checkpoint inhibition with targeted co-stimulation of adaptive immunity. DSP107 effectively blocked the CD47/SIRPα checkpoint and potentiated phagocytic uptake of cancer cells by macrophages and PMNs in vitro*.* The binding of DSP107 to CD47 also enabled the activation of 4-1BB-mediated costimulatory signaling and, thereby, potentiated anticancer T cell immune responses in vitro. Anticancer activity of DSP107 was further demonstrated in a xenograft model of DLBCL in NSG mice, providing proof of efficacy in vitro and in vivo. Finally, DSP107 treatment of cynomolgus monkeys did not associate with any toxicity, with also no impact on RBC count, and no dose-limiting toxicity detected.

DSP107 is designed to stimulate T cells by tumor-targeted activation of 4-1BB, a costimulatory receptor transiently upregulated upon MHC-TCR interaction [28]. 4-1BB has been used in various studies as a surrogate marker for tumor-reactive TILs [29], and targeting of 4-1BB with agonist antibodies and recombinant 4-1BBL fusions has been clinically evaluated [19, 25, 27, 29, 30]. However, treatment with antibodies such as urelumab, despite promising results in preclinical studies, encountered dose-limiting hepatotoxicity [20]. Such toxicity has been attributed to hyperclustering of 4-1BB due to Fc-FcγR interaction [17]. Importantly, 4-1BB signaling is only effectively activated by agonistic antibodies or by 4-1BBL when oligomerization of 4-1BB occurs, with soluble trimeric 4-1BBL being essentially inactive. In line with this, DSP107 lacks 4-1BB agonism in solution but acquires prominent 4-1BB agonistic activity upon binding to CD47. This agonism depends on the expression level of CD47 on surrounding cells, leading to activation of T cells primarily in the TME, where CD47 is overexpressed. Thus, the binding of DSP107 to tumor-overexpressed CD47 enables its 4-1BB agonistic activity and helps drive T cell immune responses. Moreover, since 4-1BB expression on immune cells is virtually absent in the circulation [17], but expressed on tumor-reactive T cells in the TME [18–20], DSP107 preferentially (re)activates T cells at the site of the tumor.

DSP107 enhanced PMN- and macrophage-mediated phagocytosis alone and when combined with RTX. The decision to phagocytose is regulated by a host of activating and inhibitory mechanisms, with CD47-SIRPα being a prominent inhibitory signal. Simply breaking this interaction is sufficient to reactivate macrophages and induce phagocytosis. For example, a F(ab’)2 preparation of a CD47 mAb, lacking an Fc-domain, was equally effective compared to a full IgG-containing antibody [31]. However, in recent studies, neither monomeric nor dimeric SIRPα increased phagocytosis alone, with an additional pro-phagocytic signal such as Fc-FcγR interaction being required for activation of phagocytosis [10, 32]. Monomeric SIRPα in our experiments also did not induce phagocytosis as a single agent. In contrast, the trimeric SIRPα present in DSP107 as a single agent enhanced phagocytosis by 5–20%, an effect probably not solely due to stronger CD47-DSP107 interaction as an increase in K_D_ was previously identified as insufficient to reactivate macrophages by monomeric or dimeric SIRPα [32]. We hypothesize that the increase in avidity combined with higher-order oligomerization and possibly raft reorganization of SIRPα-CD47 may together contribute to the DSP107-mediated phagocytosis activation. In line with this hypothesis, exosomes loaded with SIRPα induced phagocytosis of cancer cells up to 30% without a requirement for Fc [33], a level similar to that obtained in experiments with DSP107.

DSP107 combination with RTX further augmented phagocytic removal, with a statistically significant increase in macrophage and PMN-mediated phagocytosis compared to RTX alone in various cell lines and primary patient-derived blasts. Of note, this increase in phagocytosis of ~ 20% by DSP107 is in line with that reported for the CD47 mAb B6H12 in combination with RTX [31]. Notably, such in-vitro therapeutic effects translated into complete responses in relapsed or RTX-refractory lymphoma patients [3], highlighting the promise of this approach. Indeed, DSP107 proved to have equivalent activity compared to CD47 mAb or SIRPα:Fc. Thus, CD47 checkpoint inhibition by DSP107 is comparable to current state-of-the-art therapeutics.

An important concern when using CD47-targeting therapeutics is binding to erythrocytes and their potential elimination, which can lead to anemia. However, DSP107 negligibly binds to human RBCs, similar to previous reports on SIRPα-Fc fusions [10]. This phenomenon may possibly be attributed to the lack of CD47 mobility in the RBC membrane, which could prevent clustering and limit the binding of DSP107 that contains three copies of SIRPα in each molecule. Moreover, the treatment of cynomolgus monkeys with DSP107 resulted in a good safety profile. Thus, although to be further characterized in human clinical trials, DSP107 appears to have a good safety profile that may offer an advantage over CD47 antibody-based therapies.

This work supports DSP107 as a potential candidate for combinatorial therapies with opsonizing antibodies. The efficacy of the combination of CD47 blockade with antibodies directed against CD19 [34], CD20 [35] or CD70 [36] was also demonstrated in several studies. The efficacy of CD47 blocking antibodies in combination with PD-L1 antagonists has also been proven in several studies [37–39]. PD-L1 antagonists may work synergistically with the 4-1BBL domain of DSP107 to enhance anticancer T cell cytotoxicity. In fact, a combination of a 4-1BB agonist with PD-1/PD-L1 blockade increased T cell infiltration and had a prominent combination effect on tumor eradication compared to single treatments in a preclinical mouse model [40]. Additionally, TAMs accumulate in tumors after chemo and radiotherapy and initiate a wound-healing program that contributes to the suppression of T cells [41, 42]. Therefore, reactivation of TAMs after chemo and radiotherapy with simultaneous co-stimulation of T cells by DSP107 may have beneficial effects on tumor regression after treatment.

## Conclusion

**Fig. 6:**
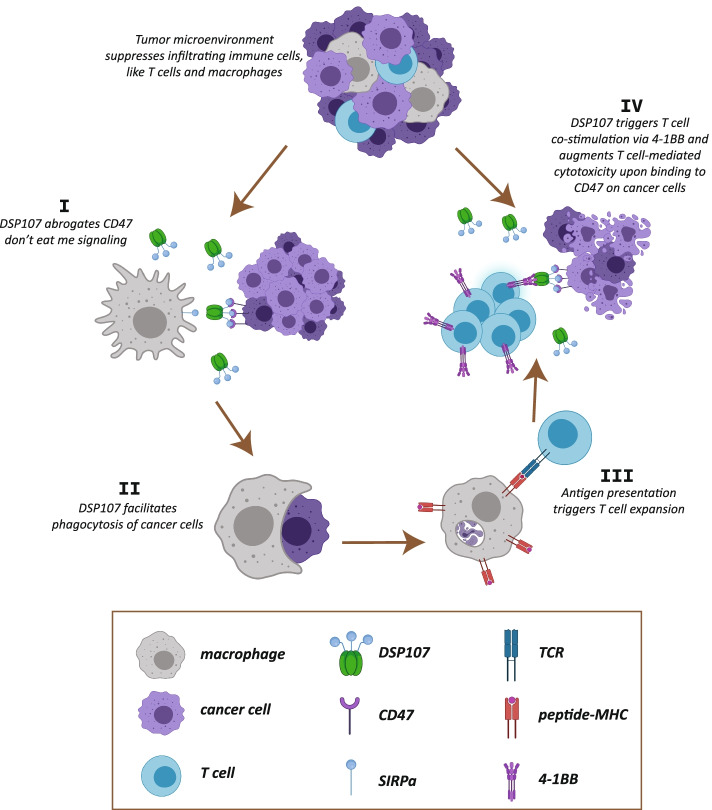
*Proposed mechanism of action of DSP107 for activation of innate and adaptive anticancer immunity.* The tumor microenvironment suppresses infiltrating immune cells, including macrophages and T cells **I**) DSP107 binds to CD47 overexpressed on cancer cells and abrogates the CD47-SIRPα interaction **II**) Consequently, macrophage-mediated phagocytosis of cancer cells is restored **III**) Upon phagocytic uptake, tumor antigens are presented to T cells in MHC, thereby triggering activation of tumor-reactive T cells **IV**) Upon recognition of cancer cells, 4-1BB is upregulated on T cells, whereupon DSP107 provides costimulatory signaling that augments antitumor T cell immunity. T cell-mediated lysis of cancer cells then further leads to phagocytic uptake of apoptotic cancer cells to continue driving the innate/adaptive anticancer immunity cycle. The figure was created with BioRender.com

DSP107 is a novel first-in-class therapeutic fusion protein with multifold immunostimulatory effects on innate and adaptive immunity. This immunostimulatory effect may, alone or together with therapeutic antibodies, serve to effectively (re)activate anticancer immunity. Based on our findings and previous publications on the same targets, we propose that DSP107 can activate several mechanisms for tumor eradication (illustrated in Fig. 6): 1) DSP107 blocks ‘don’t eat me’ signaling and promotes phagocytosis (Fig. 6, I-II), 2) DSP107 can promote antigen presentation in the context of MHC and thereby activates T cells (Fig. 6, III), 3) DSP107 co-stimulates tumor-reactive T cells via 4-1BB interaction at the tumor site, leading to additional T cell-mediated anticancer cytotoxicity (inducing phosphatidylserine exposure) (Fig. 6, IV), 4) this process, in turn, provides a feed-forward mechanism that promotes phagocytosis facilitated by DSP107-mediated CD47 checkpoint blockade (Fig. 6, I).

## Supplementary Information

**Additional file 1.** [43–45].

**Additional file 2: Video S1.** Macrophage with attached untreated cancer cell to its surface.

**Additional file 3: Video S2.** Macrophage with engulfed several SUDHL10 cells upon DSP107 treatment.

## Data Availability

Available upon request to corresponding author.

## Abbreviations

DLBCL: Diffuse Large B Cell Lymphoma
PMN: Polymorphonuclear neutrophil
ECD: extracellular domain
RTX: Rituximab
CR: Complete response
TCR: T cell receptor
MHC: major histocompatibility complex
TIL: Tumor-Infiltrating Lymphocyte
PBMCs: Peripheral Blood Mononuclear Cells
LPS: Lipopolysaccharide
IFN-γ: Interferon-γ
MCL: Mantle Cell Lymphoma
MW: Molecular weight
DSP107: Dual Signaling Protein 107
AST: Aspartate aminotransferase
ALT: Alanine transaminase
