# Supplementary material for: DSP107 combines inhibition of CD47/SIRPα axis with activation of 4-1BB to trigger anticancer immunity
Source: J Exp Clin Cancer Res. 2022 Mar 14;41:97. doi: 10.1186/s13046-022-02256-x (PMC8919572; doi:10.1186/s13046-022-02256-x)
Supplement: Supplementary file 1 — Additional file 1. [43–45]. [file 13046_2022_2256_MOESM1_ESM.zip › 13046_2022_2256_MOESM1_ESM/20220111 supplementary materials and methods_ESM (1).docx]

**Supplementary materials and methods**

**Reagents**

Mammalian expression vector pMAXX1 was purchased from Cobra Biologics and pHR_PGK_anti-CD19_synNotch_Gal4VP64 construct was a kind gift from Wendell Lim (Addgene #79125) (43). Human 4-1BB:Fc (Cat# 838-4B-100), human and mouse CD47 (Cat# 9396-CD-100 and 1866-CD-050 respectively) and PD-L1 (Cat# 156-B7-100), recombinant human ECD of SIRPα with C–terminal 6xHis-tag (Cat# 9378-SA-050) and ECD of 4-1BBL (Cat# 2295-4L-025/CF) were purchased from R&D Systems and 4-1BB (Cat# LS-G4041-100) from LSbio. Human CD47:Fc and biotinylated 41BB were obtained from GenScript (China). CD47 antibody (sequence from InhibRx, Ab.6.12 clone 2A1) with human IgG4, the SIRPα (IgV domain) IgG4 Fc fusion (sequence from TTI-622) and the 4-1BB antibody (sequence from Urelumab) were produced by GenScript (China). Myc-Tag Alexa Fluor 647 (clone 9B11) (Cat# 22335) was obtained from Cell Signaling, CD47-APC (clone CC2C6) (Cat# 323123), 4-1BB-APC (clone 4B4-1) (Cat# 309809), 4-1BBL-APC (clone 5F4) (Cat# 311505), CD45-PerCP (Cat# 304025), CD11b-Alexa Fluor-594 (clone M1/70) (Cat# 101254) antibodies and Zombie NIR^TM^ viability dye (Cat# 423105) were purchased from Biolegend. CellTrace™ CFSE (Cat# C34554) was purchased from Thermo Fisher Scientific and Incucyte® Cytolight Rapid Red Dye for Live-Cell Cytoplasmic Labeling (Cytolight Red) (Cat# 4711) from Sartorius. For IHC, CD11c polyclonal Ab (Cat# BS-1014R) was purchased from Bioss, CD8 polyclonal Ab (Cat# NBP2-29475) from NovusBio, CD4 (EPR19514) (Cat# ab183685) and FOXP-3 Ab (EPR22102-37) (Cat# ab215206) from Abcam. AnnexinV-FITC/APC was obtained from either Thermo Fisher Scientific (Cat# BMS147FI/A35110) or Immunotools (Cat# 31490013/31490016). Granulocyte-Macrophage Colony Stimulating Factor (GM-CSF) (Cat# 11343128), Macrophage - Colony Stimulating Factor (M-CSF) (Cat# 11343118), Interferon-γ (IFN-γ) (Cat# 11343537) and Interleukin 10 (IL-10) (Cat# 11340108), CD19-FITC (clone LT19) (Cat# 21270193), CD5-PE (clone LT1) (Cat# 21380054), CD11b-APC (clone MEM-174) (Cat# 21279116) were purchased from Immunotools and Lipopolysaccharide (LPS) (Cat# 916374) from Sigma-Aldrich. DSP107 was labeled with NHS-Fluorescein (5/6-carboxyfluorescein succinimidyl ester) (Cat# 46409) from Thermo Fisher Scientific and CD47 antibody was labeled with Alexa Fluor® 647 Conjugation Kit (Cat# ab269823) from Abcam where indicated. IL-8 ELISA Kit (Cat# D8000C) was purchased from R&D Systems. PBMCs were received from Sanquin, NL under agreement number NVT0465.01 or Hadassah blood bank, IL under Helsinky approval No. 0155-17-HMO. Ficoll-Paque was purchased from GE. Negative selection beads to isolate CD8+ (Cat# 130-096-495), CD3+ (Cat# 130-096-535) and CD4+ (Cat# 130-096-533) T cells were purchased from Miltenyi Biotec. NSG mice NOD.Cg-Prkdc Il2rg/SzJ, ST. 005557 were purchased from Jackson Laboratories.

**Cell lines and culture conditions**

OCI-ly3, U-2932, SUDHL4, SUDHL6, SUDHL10, SUDHL2, Ramos, Daudi, SC-1, HT-1080, and CHO-K1 cells were obtained from Deutsche Sammlung from Microorganism und Zellculturen, Braunschweig, Germany), American Type Culture Collection (ATCC) (Manassas, Virginia, US) or Gibco and cultured according to supplier’s recommendation. Cells were regularly tested by STR profiling and were mycoplasma free. HT1080.4-1BB cells were a kind gift from prof. dr. Harald Wajant (University of Wuerzburg, Germany (44).

CHO-K1.CD47 cells were generated by transducing wild-type CHO-K1 with human CD47 encoding lentivirus. Single-cell clones expressing high, medium, or low levels of membrane CD47 were generated by sorting of parental CHO-K1.CD47 cells based on the level of staining with CD47-APC Ab. Expression of 4-1BB and CD47 were determined using 4-1BB-APC or CD47-APC antibodies. To generate SC-1.scFvCD3, CHO-K1.scFvCD3 and CHO-K1.CD47.scFvCD3 cells, the scFvCD19 was replaced with scFv UCHT-1v9 in the Lentiviral synNotch receptor construct pHR_PGK_anti-CD19_synNotch_Gal4VP64 using Gibson assembly according to manufacturer’s protocol. Lentiviral particles were produced as previously described (45) and used to transduce SC-1, CHO-K1 or CHO-K1.CD47 cells. Cells were sorted based on Myc-Tag expression located on the N-terminus of the scFvCD3, using Myc-Tag AF647 using Sony SH800s sorter. All cells and cultures were maintained at 37 °C at 5% CO_2_.

**Isolation and culture of immune cells and primary blasts**

Peripheral blood mononuclear cells (PBMCs) were isolated by density gradient centrifugation from whole blood or buffy coat material. CD3^+^, CD4^+^ or CD8^+^ T-cells were isolated by negative selection with magnetic beads. Monocytes were isolated by seeding PBMCs in 6-well plates and removing non-adherent PBMCs after 2 h of incubation at 37 °C. Adherent monocytes were differentiated to M0 macrophages with GM-CSF/M-CSF (50 ng/mL each) for seven days. M1 macrophages were generated by treatment of M0 macrophages with LPS (100 ng/mL) and IFN-γ (20 ng/mL) for two days, whereas M2 macrophages were generated by treatment with IL-10 (50 ng/mL) for two days. Granulocytes were isolated from the blood of healthy volunteers from the pellet obtained after density gradient centrifugation, followed by red blood cell (RBC) lysis using ammonium chloride lysis solution (150 mM NH_4_Cl, 10 mM NaHCO3, 1.2 mM EDTA). Primary Mantle Cell Lymphoma (MCL) blasts were obtained from anonymised waste material and phenotyped using CD19/CD5 flow cytometry. Autologous macrophages were obtained by isolation of monocytes and macrophage differentiation as described above. All the procedures with primary material were performed in accordance with International Ethical and Professional Guidelines (the Declaration of Helsinki and the International Conference on Harmonization Guidelines for Good Clinical Practice).

**DSP107 production and characterization**

The coding sequences of the ECDs of human SIRPα and 4-1BBL (Table S1) were codon-optimized and the fused DNA sequence was synthesized and directionally cloned into the pMAXX1 vector. The nucleotide sequence was verified by Sanger sequencing methodology (GeneArt, Germany). DSP107 was produced using CHO-S cells transfected with the pMAXX1-DSP107 vector, whereupon a high expressing CHO-S.DSP107 clone was selected. Production of DSP107 was performed using a fed-batch process. Cell culture media was harvested and clarified by depth filtration, and DSP107 was purified by diafiltration (DF) followed by anion exchange chromatography, protein concentration, and buffer exchange into PBS pH 7.4. A dual-binding ELISA assay was used to quantitatively analyze the simultaneous binding of SIRPα to recombinant CD47 and 4-1BBL to recombinant 4-1BB. Briefly, flat bottom 96-well plates were pre-coated with recombinant hCD47-Fc overnight at 4°C, washed (PBS-0.05% Tween), and blocked with Diluent Buffer (DB) (1% BSA/0.05% PBS-T) for 1hr at room temperature (RT). Following three additional washing steps, serial dilutions of DSP107 in DB were added and incubated for 2hr at RT, washed three times, and incubated with h41BB-Biotin for 1hr at RT. After three washes, Streptavidin-HRP (1:10,000 in PBS) was added and incubated 1hr at RT and light protected. Following three additional washes, the ELISA was developed using Ultra-TMB according to the manufacturer’s recommendations and analyzed at 450nm. A standard curve was plotted using a 4PL model, and EC50 was calculated by Graphpad Software.

To evaluate the molecular weight (MW)**,** DSP107 was loaded on a Superdex Increase 200 gel filtration column (GE Healthcare) and run at a flow rate of 0.8 mL/min with 10 mM K_3_PO_4_ pH 8.0 + 150 mM NaCl as mobile phase. Detection was performed using a combination of UV, MALS, and Refractive index (RI), using ÄKTA^™^ Pure + miniDAWN™ TREOS® + OPTILAB® T-reX™.

**Binding assays**

Bio-layer interferometry (BLI) Blitz® (ForteBio) was performed using anti-human IgG-Fc Capture Biosensors pre-loaded with CD47:Fc or 4-1BB:Fc (1 µg/mL), followed by DSP107 or MOCK-DSP incubation and subsequent wash. Binding kinetics of DSP107 for human and mouse CD47 and 4-1BB was assessed using Surface Plasmon Resonance (SPR) on the Biacore T100 biosensor system (GE Healthcare, USA; study performed by the Biointeraction SPR Facility, Bar Ilan University, Israel) with a Series-S sensor chip CM5. Anti-hIgG antibodies were coupled to the channels using a standard amine-coupling protocol. PD-L1:Fc (negative control), CD47:Fc and 4-1BB:Fc were coupled in running buffer (10 mM HEPES pH7.3, 150 mM NaCl, 3 mM EDTA, 0.05% Tween20), whereupon DSP107 (0.0253-52.5 µg/mL) was passed over the channels. Binding parameters were evaluated by BiaEvaluation software v.3.0.2 (GE).

DSP107 was labeled using NHS-esther-FITC at molar ratio 10:1. SUDHL10 or HT-1080.4-1BB cells were incubated with DSP107-FITC (2.5 µg/ml) for 1h at 4°C. Doublet formation of CFSE-stained CHO-K1.CD47 and Cytolight Red-stained HT1080.4-1BB were assessed by co-incubation of 5x10^4^ cells with/without DSP107 (5 µg/mL) for 45 minutes at room temperature (RT). Where indicated, cells were preincubated with CD47 mAb (10 µg/ml) or soluble 4-1BBL (s4-1BBL) (5 µg/mL) and analyzed by flow cytometry (BD Accuri C6). For DSP107 binding to RBCs, 100µL of 1:500 diluted whole blood (~1x10^6^ RBCs) mixed with 1x10^5^ PBMCs and 1x10^5^ CPD-stained SUDHL4 cells was incubated with DSP107 (0-50 μg) or AF647-labelled CD47 mAb InhibRx. Cells were incubated with 4-1BBL-APC Ab, for DSP107 detection and CD45-PerCP for immune cell identification.

**Table S1.** SIRPα-4-1BBL Sequences

| hSIRPα ECD | EEELQVIQPDKSVLVAAGETATLRCTATSLIPVGPIQWFRGAGPGRELIYNQKEGHFPRVTTVSDLTKRNNMDFSIRIGNITPADAGTYYCVKFRKGSPDDVEFKSGAGTELSVRAKPSAPVVSGPAARATPQHTVSFTCESHGFSPRDITLKWFKNGNELSDFQTNVDPVGESVSYSIHSTAKVVLTREDVHSQVICEVAHVTLQGDPLRGTANLSETIRVPPTLEVTQQPVRAENQVNVTCQVRKFYPQRLQLTWLENGNVSRTETASTVTENKDGTYNWMSWLLVNVSAHRDDVKLTCQVEHDGQPAVSKSHDLKVSAHPKEQGSNTAAENTGSNERNIY |
| --- | --- |
| H4-1BBL ECD | ACPWAVSGARASPGSAASPRLREGPELSPDDPAGLLDLRQGMFAQLVAQNVLLIDGPLSWYSDPGLAGVSLTGGLSYKEDTKELVVAKAGVYYVFFQLELRRVVAGEGSGSVSLALHLQPLRSAAGAAALALTVDLPPASSEARNSAFGFQGRLLHLSAGQRLGVHLHTEARARHAWQLTQGATVLGLFRVTPEIPAGLPSPRSE |

**Table S2.** DSP107 Binding Affinity Constants (K_D_) to Human, Cynomolgus Monkey and Mouse CD47 and 4-1BB

| **Counter Receptor** | **K_D_ (nM)** | | |
| --- | --- | --- | --- |
|  | **Mouse** | **Monkey** | **Human** |
| **CD47** | 0 | 1.75 nM | 1.17 nM |
| **4-1BB** | 0 | 0.34 nM | 0.7 nM |

**Supplementary Figures**

**Figure S1. DSP107 dual ELISA and binding to cells.** **A)** Dual binding ELISA of three DSP107 batches, **B)** Expression level of CD47 and 4-1BB on HT1080.wt and HT1080.4-1BB cell lines, **C)** Binding of DSP107 to HT1080.4-1BB with 4-1BBL blocking alone, **D)** Expression of 4-1BB on various PBMC components, **E)** Expression of CD47 on various PBMC components,**F)** Binding of DSP107 to various PBMCs alone (left graph) and after blocking with CD47 mAb (right graph).

**Figure S2. Expression of polarization markers and 4-1BB on macrophages.**

**A)** Confirmation of polarization to M1 and M2c subtype by surface marker expression (CD86 and CD163), **B)** Representative histograms of 4-1BB expression on M1 and M2 macrophage subtypes, **C)** Summary of 4-1BB expression on M1 and M2 macrophages from three donors

**Figure S3. Trogocytosis of cancer cells by PMNs depends on blocking CD47-SIRPα by DSP107**. **A)** Cooperativity index of PMN-mediated trogocytosis of B cell lines. Cooperativity index was calculated using equation: trogocytosis by single DSP107 treatment + trogocytosis by single RTX treatment)/ combination treatment **B)** 4-1BB is not expressed on healthy PMNs, **C)** Treatment of SUDHL-4 and U2932 cell lines with DSP107 alone does not trigger significant increase in PS exposure,

**Figure S4. Induction of IL-8 secretion by DSP107 is dependent on 4-1BB and cross-linking via CD47**. **A)** Induction of IL-8 secretion by HT1080.wt and HT1080.4-1BB cells treated with DSP107 on plate-bound CD47, **B)** Expression level of CD47 in CHO-K1 clones, **C)** Induction of IL-8 secretion by HT1080.4-1BB triggered with 4-1BB antibodies and DSP107 with plate-bound CD47.

**Figure S5. DSP107 treatment did not negatively impact mouse spleen or weight.** **A)** Scheme of DSP107 treatment schedule of NSG SUDHL6 mouse model, **B)** Mouse weight without and with DSP107 treatment, **C)** Spleen weight without and with DSP107 treatment, **D)** CD4+ T cell infiltration in the tumor, **E)** FoxP3 staining of tumor slides, **F)** Quantitative summary of infiltration of CD4+ and FoxP3 cells (5 mice were included in PBMC group and 6 mice in PBMC+DSP107 group)

**Video S1. Macrophage with attached untreated cancer cell to its surface.**

**Video S2. Macrophage with engulfed several SUDHL10 cells upon DSP107 treatment.**
