# Supplementary figures and images for: DSP107 combines inhibition of CD47/SIRPα axis with activation of 4-1BB to trigger anticancer immunity
Source: J Exp Clin Cancer Res. 2022 Mar 14;41:97. doi: 10.1186/s13046-022-02256-x (PMC8919572; doi:10.1186/s13046-022-02256-x)

**Figure S2**

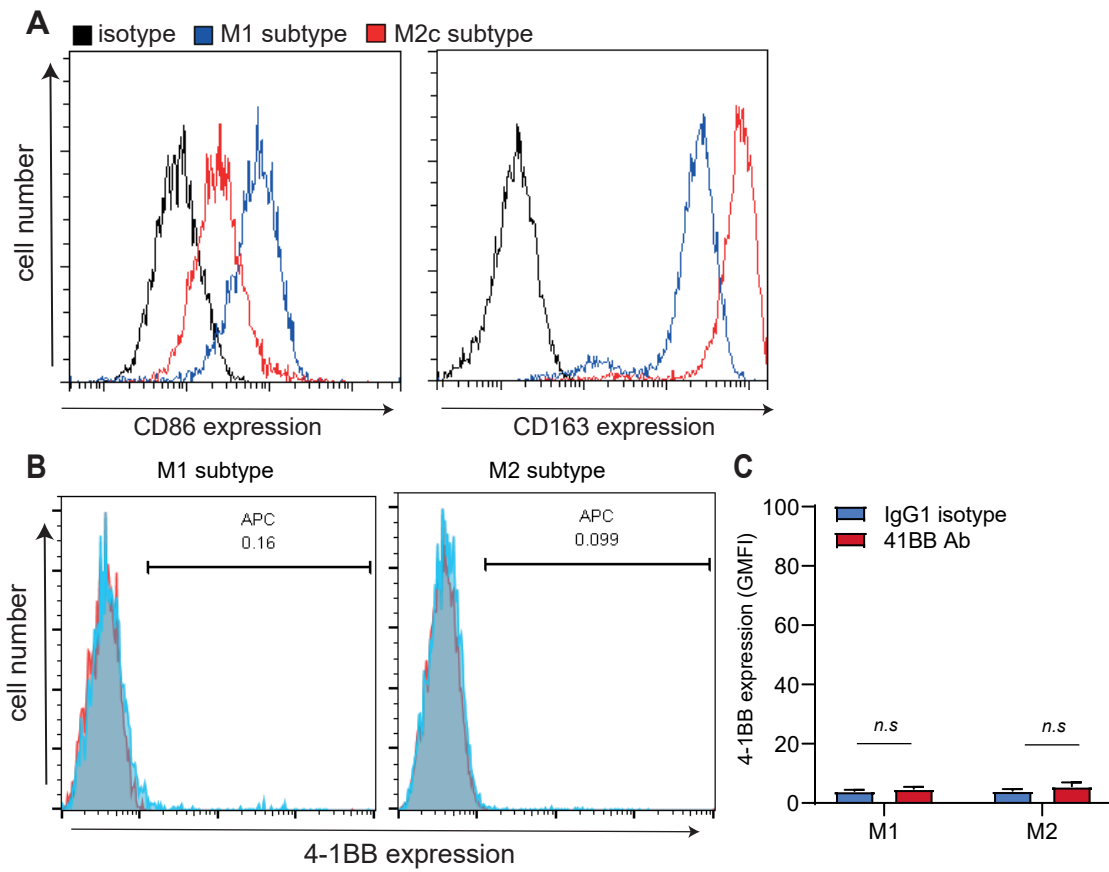

Supplement: Supplementary file 1 — Additional file 1. [43–45]. [file 13046_2022_2256_MOESM1_ESM.zip › 13046_2022_2256_MOESM1_ESM/20210730 Figure S2.pdf]

Figure S1

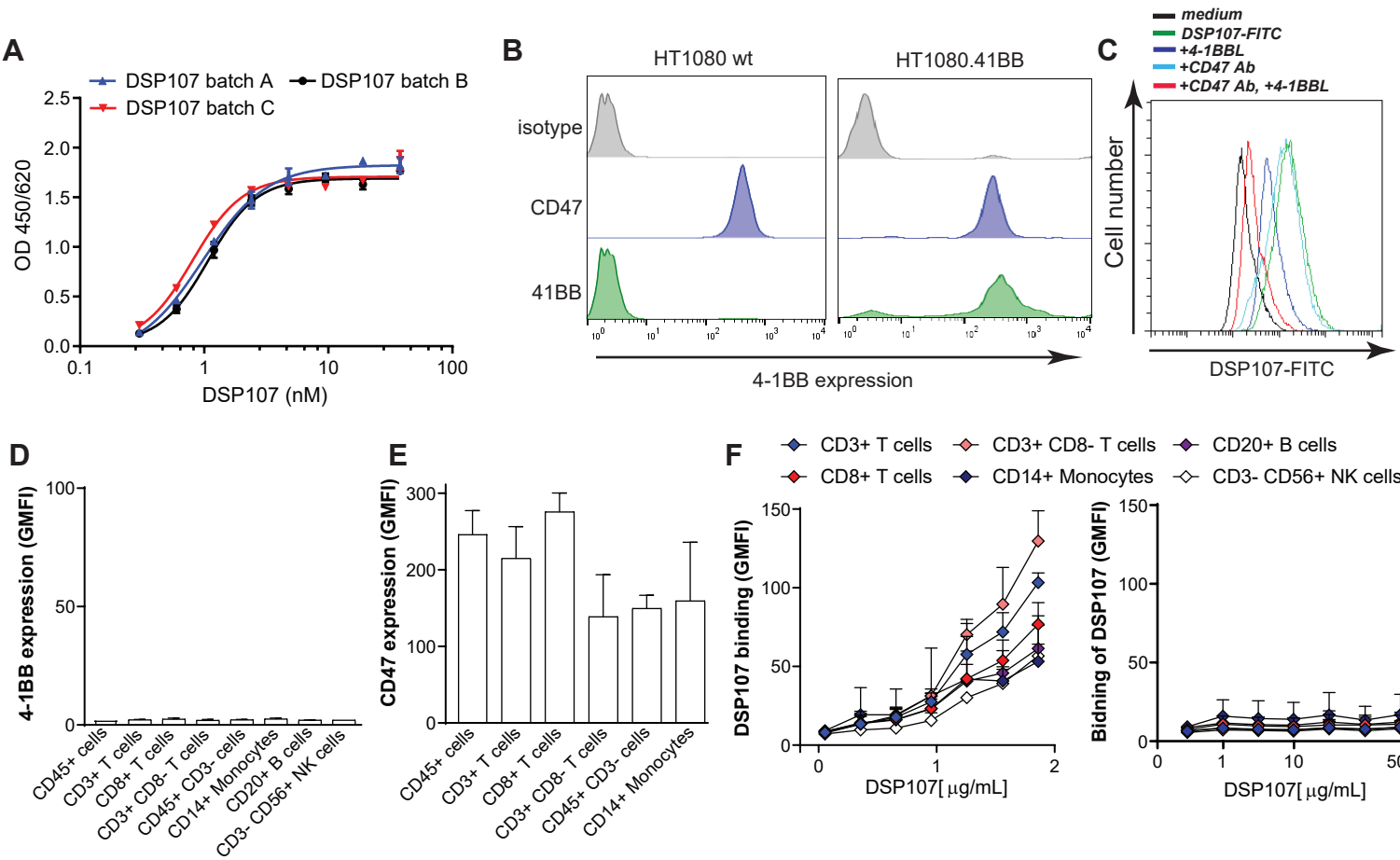

Supplement: Supplementary file 1 — Additional file 1. [43–45]. [file 13046_2022_2256_MOESM1_ESM.zip › 13046_2022_2256_MOESM1_ESM/20210831 Figure S1.pdf]

Figure S3

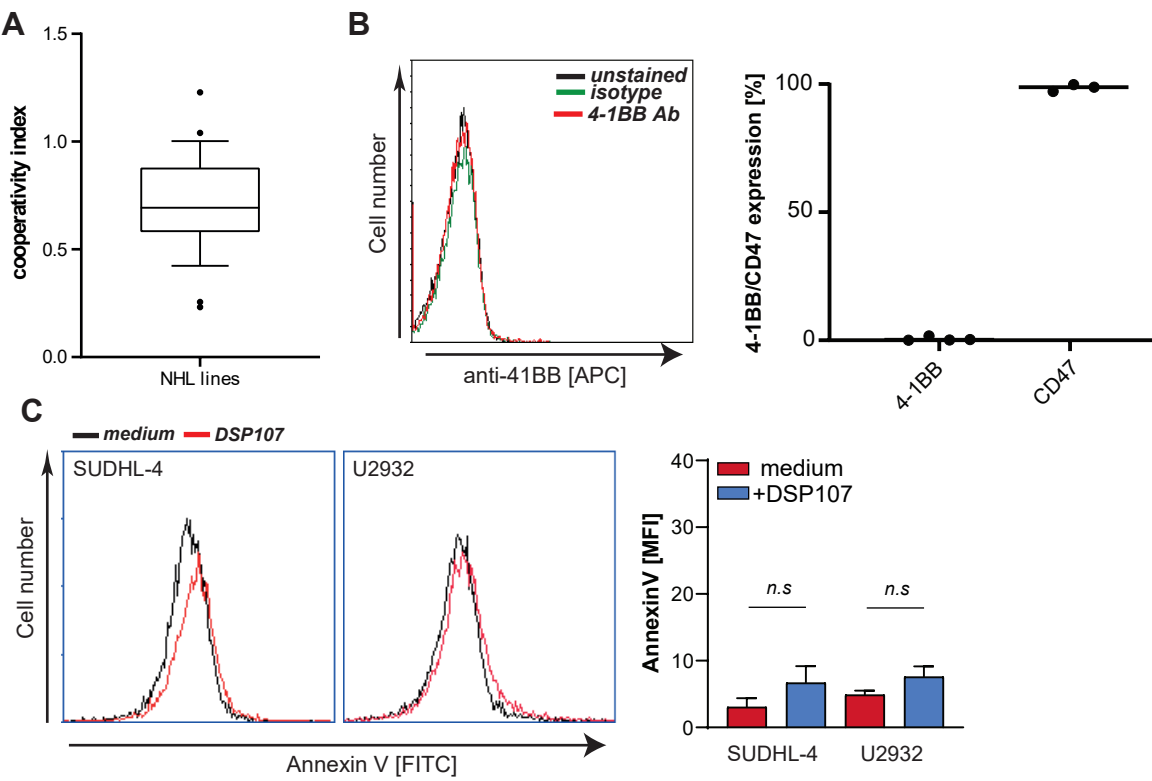

Supplement: Supplementary file 1 — Additional file 1. [43–45]. [file 13046_2022_2256_MOESM1_ESM.zip › 13046_2022_2256_MOESM1_ESM/20210831 Figure S3.pdf]

Figure S4

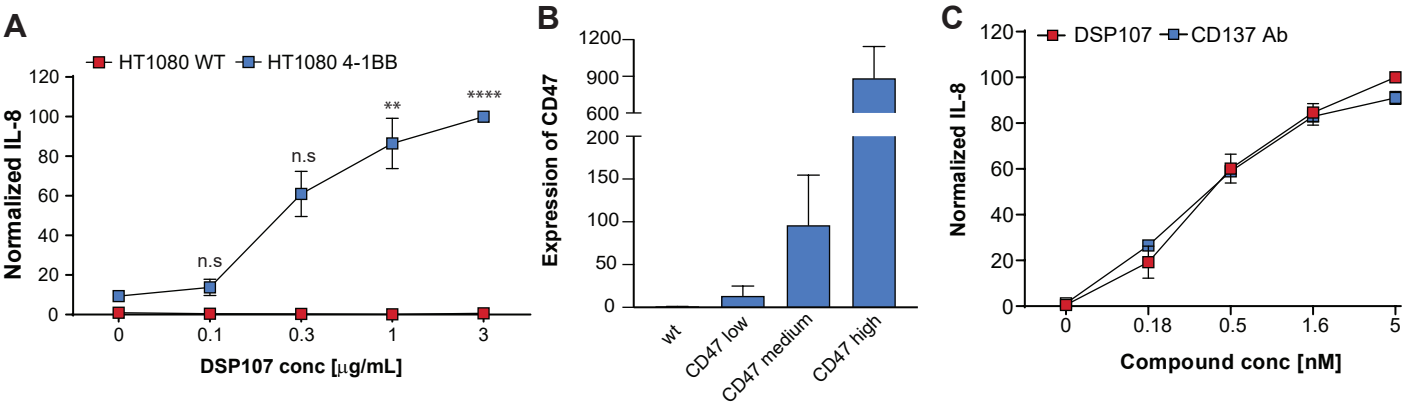

Supplement: Supplementary file 1 — Additional file 1. [43–45]. [file 13046_2022_2256_MOESM1_ESM.zip › 13046_2022_2256_MOESM1_ESM/20210831 Figure S4.pdf]

Figure S5

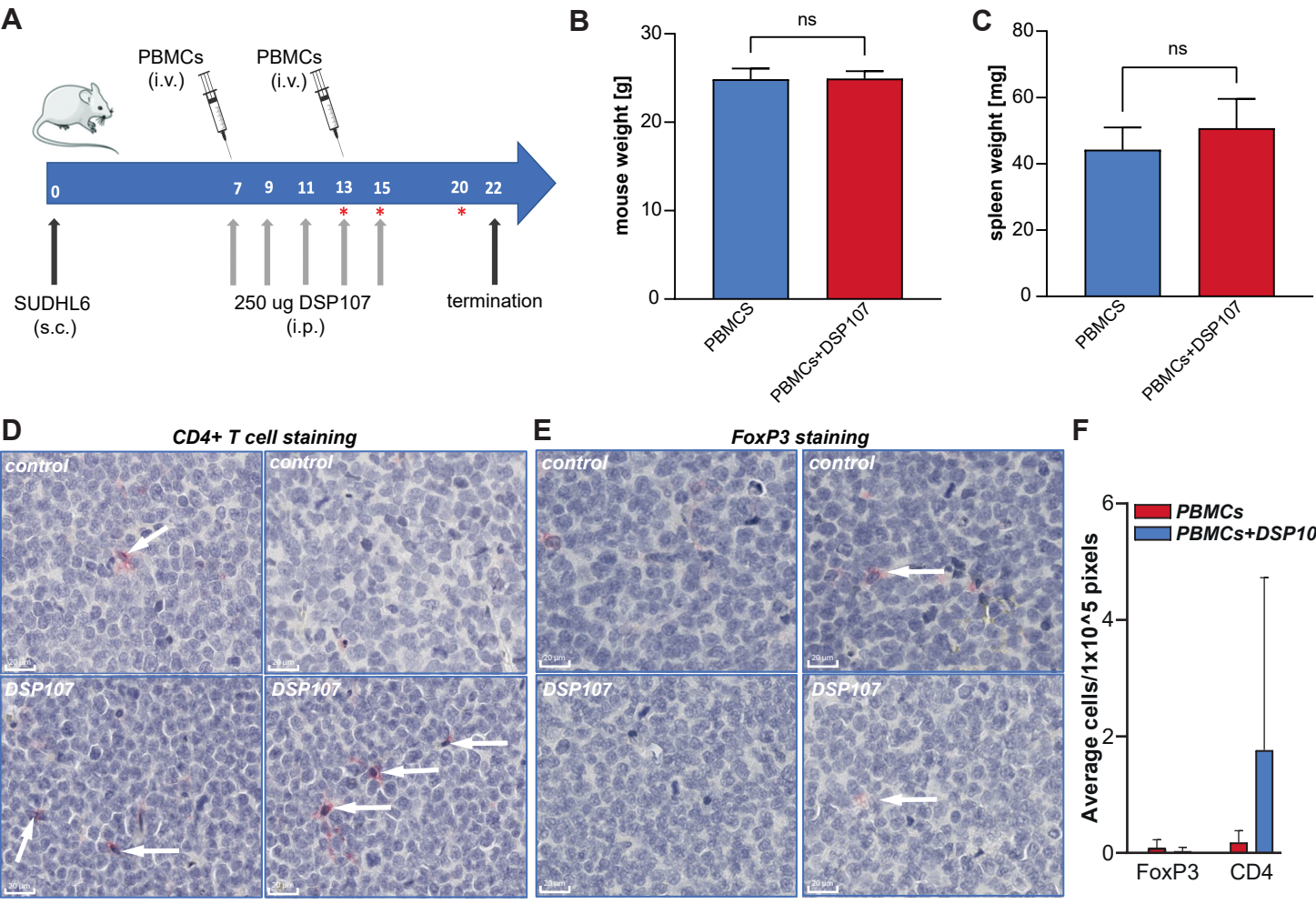

Supplement: Supplementary file 1 — Additional file 1. [43–45]. [file 13046_2022_2256_MOESM1_ESM.zip › 13046_2022_2256_MOESM1_ESM/20220114 Figure S5.pdf]
